# Parallel or clump-wise serial? A reinterpretation of feature-search mode

**DOI:** 10.3758/s13414-026-03315-7

**Published:** 2026-07-30

**Authors:** Dock H. Duncan, Jan Theeuwes

**Affiliations:** 1 Department of Experimental and Applied Psychology, Vrije Universiteit Amsterdam, Van der Boechorststraat 7, 1081 BT Amsterdam, The Netherlands; 2 Institute for Brain and Behavior Amsterdam (iBBA), Amsterdam, The Netherlands; 3 Centre for Human Brain Health, School of Psychology, University of Birmingham, Birmingham, UK; 4 Department of Psychology and Behavioral Sciences, Zhejiang University, Hangzhou, China; 5 Mind, Brain and Behavior Research Center (CIMCYC), University of Granada, Granada, Spain

**Keywords:** Attentional capture, Visual search, Feature search mode, Singleton detection mode, Clump-wise search, Serial search, Parallel search, Search slopes

## Abstract

**Abstract:**

A central debate in visual attention concerns whether selection is guided primarily by top- down control or by bottom-up salience. In the additional singleton task, the absence of attentional capture by a distractor is often attributed to the adoption of feature-search mode. An alternative account proposes that these displays instead partly induce serial search, which naturally limits distractor interference. The present study tested this by linking two diagnostics of search dynamics, set size effects and two target compatibility, within the same paradigm. Participants searched for a predefined target among heterogeneous non-targets while set size and the presence of a salient color singleton distractor were manipulated. Across two experiments, search showed reliable display size effects and responses were faster when two targets were present than when only one target was present. At larger display sizes no target compatibility effect was observed. At the same time the distractor did not capture attention and instead produced a distractor benefit. Together, these findings indicate that displays commonly assumed to induce the feature-search mode partly require serial selection. The absence of attentional capture therefore reflects sequential serial processing rather than top-down suppression.

**Open practices statement:**

All experiment code, analysis code, and anonymized trial-level participant data are available via the Open Science Framework at: osf.io/g6avc. None of the experiments herein were preregistered.

## Introduction

Over the past 30 years, a central debate in visual attention has been whether selection is mainly guided by our goals (top-down control) or whether salient events in the environment automatically attract attention (bottom-up salience). For example, we may look at an ambulance because we intend to, but its flashing lights may also capture attention involuntarily (for reviews, see Egeth & Yantis, 1997; Liesefeld et al., 2024; Luck et al., 2021; Theeuwes, 2010, 2023a, 2025, 2026; Wolfe, 2021). When salient but irrelevant items draw attention and slows responses, this is typically described as attentional capture (Theeuwes, 1991, 1992).

Much of this debate has relied on the additional singleton task (Theeuwes, 1991, 1992), where participants search for a salient target singleton while ignoring an even more salient yet irrelevant singleton distractor. The classic result is slower reaction times when the distractor is present than when it is absent, suggesting the most salient item captures attention (Theeuwes, 2010). Bacon and Egeth (1994) proposed that capture depends on search strategy: if observers adopt a singleton detection mode (looking for anything that stands out), distractors capture attention; if task design induces feature-search mode (searching for specific target features, e.g., by using heterogeneous non-target shapes), capture can be reduced.

A related view is the signal suppression account (Gaspelin et al., 2015; Gaspelin & Luck, 2018b; Gaspelin et al., 2025; Sawaki & Luck, 2010) which holds that salient items generate strong bottom-up signals but these signals can be proactively suppressed by top-down inhibition, but only when search is guided by features, not salience. While the dominant view holds that, in feature-search mode, observers can prevent attentional capture through top-down control (Bacon & Egeth, 1994; Folk et al., 1992; Gaspelin & Luck, 2018b; Leber & Egeth, 2006; Luck et al., 2021; Stilwell & Gaspelin, 2021), alternative accounts argue that “feature search mode” may not be a voluntary strategy at all. Instead, it may reflect a display-induced shift toward a more serial, or clump-wise, search when the target is not salient enough to pop out (Liesefeld et al., 2025; Liesefeld & Müller, 2020; Theeuwes, 1994, 2004, 2010, 2023a, b).

Crucial to this debate is the concept of search slopes – the observation that increasing the number of items in a display will slow search under certain conditions (resulting in a slope between the two search conditions), but not others (resulting in flat slopes; Treisman & Gelade, 1980). Theeuwes (2004) showed that, even with heterogeneous shapes that would typically require feature-search mode to identify the target, the distractor still captured attention at larger set sizes of 12 and 20 items. As the number of elements in the display increased, the target became sufficiently locally salient to be detected through parallel search. Consistent with this interpretation, search slopes were flat, indicating parallel search. Theeuwes then claimed that when the target is less locally salient, search becomes more serial (resulting in steeper search slopes) and capture disappears. This suggests that apparent “search modes” may often arise from display properties that change how attention is deployed. Conversely, Leber and Egeth (2006) utilized search slopes to demonstrate that search strategies can be “set” via training with implications for distractor processing. Using a training/testing blocked experimental design, Leber and Egeth demonstrated that participants could be induced into certain search strategies by training in either parallel or serial search trials. Subsequently, in a testing phase using parallel search displays (searching for a circle among homogeneous square non-targets; Theeuwes 1992), that could vary between five and nine search items, they demonstrated participants trained in feature search trials showed no capture by the distractor, leading them to conclude that top-down search strategies could be used to override capture. Importantly, they additionally claimed that this suppression occurred while search was parallel as search slopes were consistently flat in both groups – indicating that the target was detected equally easily in large and small set sizes.

Recently, de Waard and Theeuwes (2026) provided strong evidence that what is typically labeled as “the feature-search mode” reflects serial, clump-wise processing by adapting the two-target compatibility method developed by Han and colleagues (Jeong et al., 2025; Lee et al., 2021). In this method, some visual search trials include two targets instead of one. These targets can map onto the same response (compatible trials) or onto different responses (incompatible trials). In de Waard and Theeuwes’s study, feature-search displays produced virtually no compatibility effects, consistent with the idea that observers respond to the first target encountered during search while other items, including the second target, are not processed to influence response selection; importantly, under these same conditions the salient distractor also failed to capture attention. Also, two-target displays yielded much faster search times than one-target displays, suggesting a serial self-terminating search process, in which the response was made as soon as the first target was found.

Using this same technique, one experiment by de Waard and Theeuwes (2026) used displays similar to the classic additional singleton task, allowing participants to search for the target in parallel. The results showed the classic attentional capture effect: responses were slower when a singleton color distractor was present than when it was absent, regardless of whether the display contained one or two targets. Crucially, however, a robust compatibility effect was observed, providing convincing evidence that search was performed in parallel across the display. In addition, two-target displays produced slower responses than one-target displays, suggesting interference caused by the presence of two targets.

While compelling, de Waard and Theeuwes (2026) did not provide direct evidence that search was serial because display size was not manipulated, and so it remains unclear how target compatibility effects would map onto traditional search slope manipulations. The present study aimed to link two diagnostics – search slopes and two-target compatibility – within the same task and displays. We tested whether feature-search conditions produce non-zero search slopes together with little or no compatibility effects. This would provide direct evidence that the absence of two-target compatibility is the result of serial (clump-wise) search. Using search displays that are commonly assumed to elicit feature search (copied from Stilwell & Gaspelin, 2021), we adapted the two-target design of de Waard and Theeuwes (2026) to also examine search slopes. Participants viewed search arrays containing one or two targets, and on half of the trials a uniquely colored singleton distractor was present. We varied set size to estimate search slopes and to compute two-target compatibility effects. The central question was whether the degree of serial search suggested by set-size functions is matched by the extent of simultaneous target processing as indexed by compatibility.

## Experiment 1

Experiment 1 used the same displays and code as was used in de Waard and Theeuwes (2026). This was in turn an exact replica of the displays employed by Stilwell and Gaspelin (2021) with the exception that display size was varied between sizes 6 and 10.

### Methods

#### Participants

Based on the effect sizes observed in Experiment 2 of de Waard and Theeuwes (2026), we chose to recruit 40 participants, thereby giving us an 80% chance to observe effect sizes as low as *d* = 0.45.

Forty participants between 19 and 40 years old (mean age = 28.4 years) took part in the study through Prolific (Palan & Schitter, 2018). In order to qualify for this experiment, participants needed to report normal or corrected-to-normal vision, no color blindness, and have participated in at least 15 online experiments in the past with an acceptance rate of 100%. Participation took approximately 45 min and participants were paid £6.75.

All participants gave informed consent, and all methods were performed in accordance with the Declaration of Helsinki. The experiment was approved by the Ethics Committee of the Faculty of Behavioral and Movement Sciences of the Vrije Universiteit Amsterdam.

#### Stimuli

Because this was an online experiment, exact screen dimensions varied per participant. Item sizes are thus reported in pixel size. We used the same shape stimuli as Stilwell and Gaspelin (2021). The experiment was created in OpenSesame (Mathôt et al., 2012) and hosted on JATOS (Lange et al., 2015).

#### Procedure

**Fig. 1:**
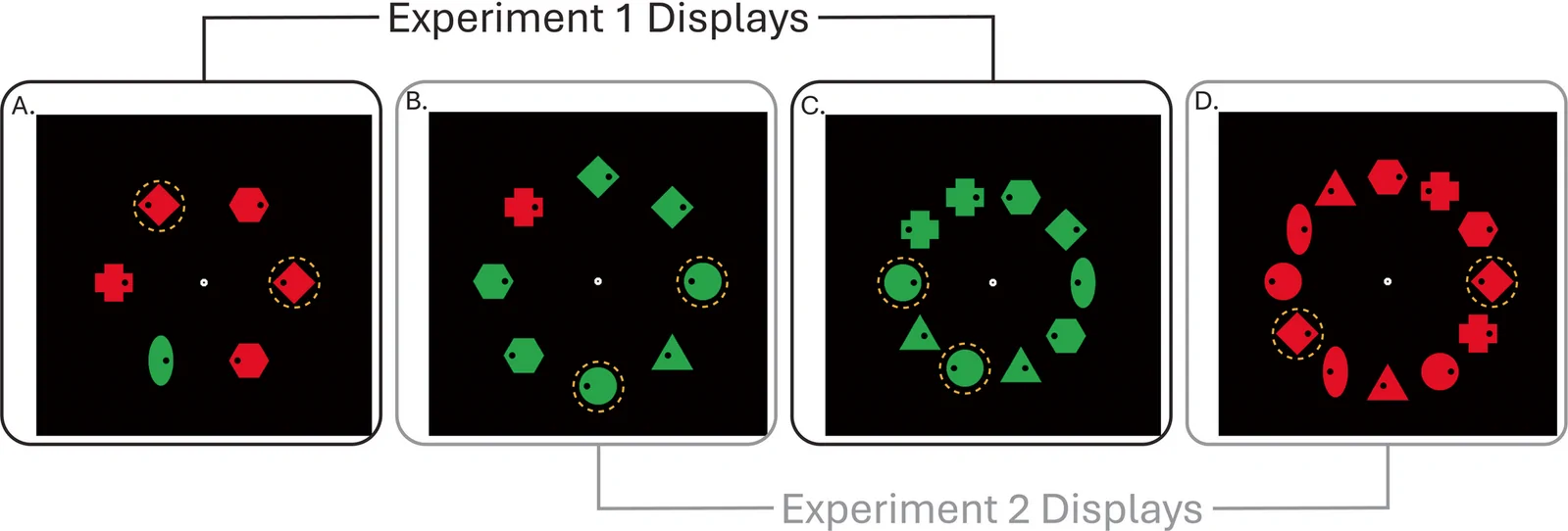
Example experiment displays used in Experiments 1 and 2. Note that all examples show two-target trials; the single-target condition (1/3 of trials) is not shown. In both experiments, participants were assigned a certain target shape (either diamond or circle) and color (either red or green) pair which remained their search target for the entire experiment session, counterbalanced across participants. Experiment 1 used six or ten item displays while Experiment 2 used eight and 12, with an even number of trials each. The dotted yellow circles were not present in the actual experiment and are used here only to direct attention to the two targets in each display. (**A**) An example six-item search display used in Experiment 1 for a participant searching for a red diamond. Shown are two compatible search targets at a distance of two away from one another. Additionally, a green color singleton distractor is present (on 50% of trials). (**B**) An example eight-item search display used in Experiment 2 for a participant searching for a green circle. Shown are two compatible search targets a distance of two away from one another. A red color singleton distractor is additionally present. (**C**) An example ten-item search display used in Experiment 1 for a participant searching for a green circle. Shown are two incompatible targets at a distance of two away from one another. (**D**) An example 12-item search display used in Experiment 2 for a participant searching for a red diamond. Shown are two incompatible targets a distance of five apart from one another

The experiment began by assigning each participant a search target. Search targets were either circles or diamonds (counterbalanced across participants) which were either red or green (also counterbalanced). The search target’s shape and color never changed once assigned and remained constant throughout the experimental session. Trials began with the presentation of a fixation dot on a black background (RGB 0,0,0) for a randomly jittered interval between 900 and 1,000 ms. Next, a display of either six or ten heterogeneous shapes appeared on the screen arranged equally spaced around a 200-px radius imaginary circle (Fig 1). The experiment was designed such that exactly half of the trials would feature six items, and half ten. Depending on what color the search target was, the displayed shapes were all either colored red (RGB 255,0,0) or green (RGB 14,198,0). Furthermore, in half of all trials one of the non-target shapes was converted into a distractor by changing its color to the non-target color, thereby making it a color singleton. The ten shapes used were the two potential target shapes – circle (82-px diameter) and diamond (94-px wide) – as well as crosses (83-px wide), hexagons (89 × 77 px), ovals (56 × 112 px), and triangles (92 × 80 px; stimuli files are available here: https://osf.io/jwbgv). On each trial, the non-target shapes were a random collection of alternative shapes with the condition that no shape appeared more than twice on a single display. Because the search target had a consistent shape and color dimension, and because it was presented in heterogeneous displays of non-matching shapes, participants in this experiment could be said to have been engaged in the feature-search mode of visual search (Bacon & Egeth, 1994; Lamy & Egeth, 2003).

Every shape in a display contained a black dot (14-px diameter) offset to the left/right of the center of the shape. Each participant’s job was to identify their assigned target shape, and report whether it contained a dot displaced to the left or right of center. Responses were given using the left and right direction buttons to indicate the dot’s location. On each trial, there could either be a single target (1/3 of trials) or two target shapes embedded in the display (2/3 of trials). On two-target trials, target placement was controlled such that targets never appeared directly adjacent to each other in the ten-item condition. In the six-item condition this was not possible. Furthermore, targets and distractors were never adjacent to one another in the ten-item condition but could be in the six-item condition. Additionally, when two targets were presented, they held dots at either the same location within their respective shapes (1/2 of two-target trials) or at opposite locations. If both targets held dots on the same side (thereby encouraging the same response) then these trials were labeled compatible trials. If they did not (thereby evoking opposite responses), then they were labeled incompatible. Note that on incompatible trials, regardless of what answer participants provided, the trial would be labeled as correct. Once the search display was shown, participants had up to 2,500 s to provide a response, after which feedback was given in the form of a smiling (300 ms) or sad (750 ms) emoticon, or the message “Too slow!” (1,000 ms) if the trial timed out.

Each experimental session began with comprehensive instructions, followed by 30 practice trials (discarded from the main analysis) which the participant needed to be at least 90% accurate on, or else a new round of 30 practice trials would begin. Participants then began 12 blocks of 60 trials each before the experiment’s conclusion.

### Results

Trials with reaction times 2.5 standard deviations away from the individual participant’s mean reaction time were excluded from all analyses (4.1% of total data). Because two-target incompatible trials were always recorded as correct, and to ensure fair comparisons, incorrect trials were also included in all subsequent analyses. It is known that reaction time data distributions often violate normality (Duncan et al., 2026; Palmer et al., 2011). Appropriate non-parametric tests are used below whenever called for.

**Fig. 2:**
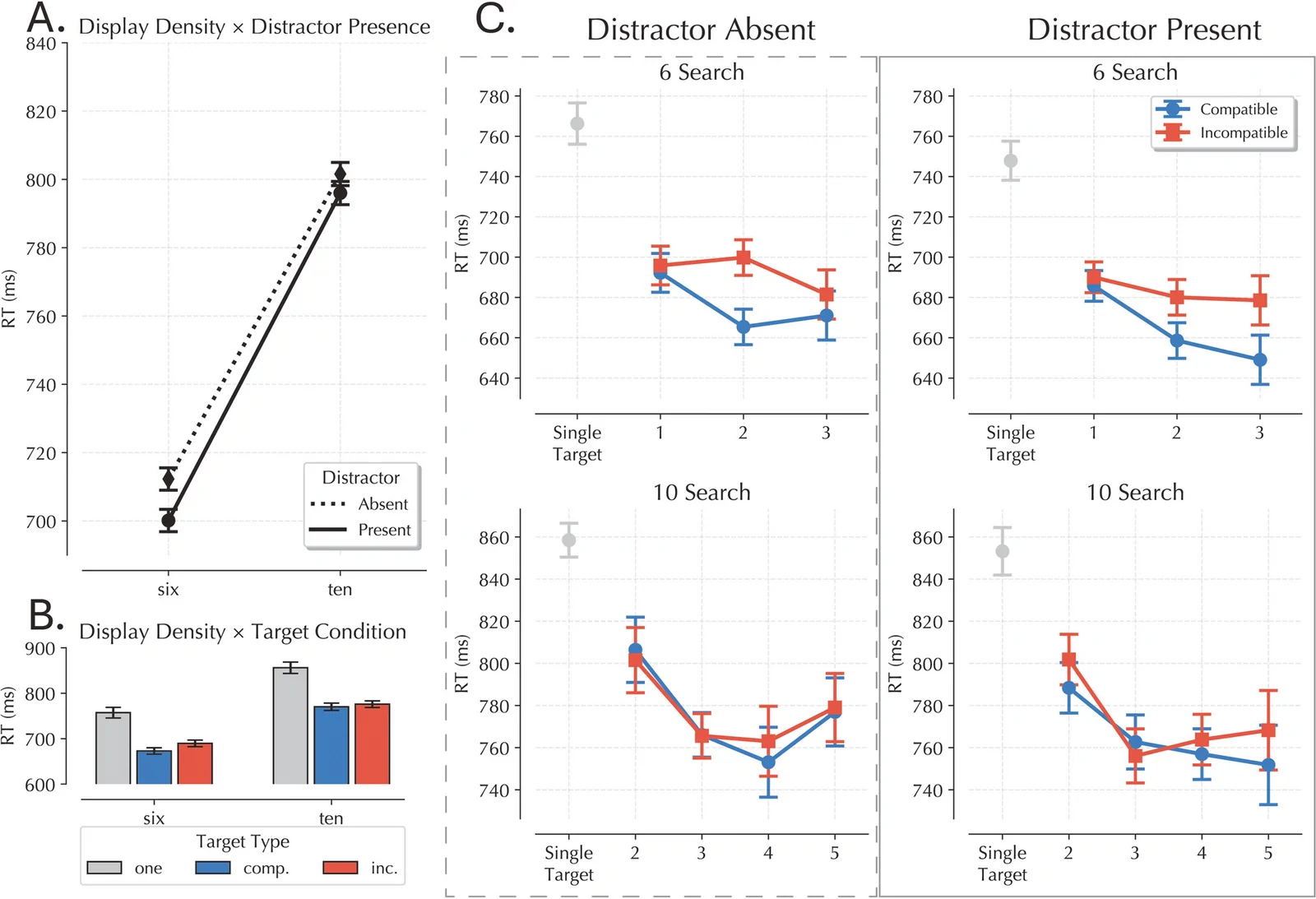
Results from Experiment 1 where search arrays randomly swapped between six- and ten-item displays. (**A**) interaction between number of search items (six or ten) and distractor condition (present or absent). Targets were detected reliably faster in six-item arrays than in ten-item displays (~93 ms), but no interaction with distractor presence was found. (**B**) Comparison of target conditions across the two possible display sizes. In the legend: one indicates single-target trials, comp. indicates two compatible target trials, inc. denotes two incompatible target trials. Single-target conditions were reliably slower than dual-target conditions, and incompatible target trials were slower than compatible trials in six-item displays only. (**C**) Target-target distance effects separated by display size (six- and ten-item search displays) and distractor-presence conditions.* Note:* A repeated-measure ANOVA taking target-target distance and compatibility as well as distractor conditions as factors separately for six- and ten-item search trials observed no reliable interactions. Note the differences between Y-axes, matched within rows but not columns. All error bars are within-subject 95% confidence intervals (Cousineau, 2005; Morey, 2008)

A 2 x 2 repeated-measure analysis of variance (rmANOVA) taking display size (six- and ten-item displays) and distractor condition (present/absent) revealed significant main effects of both display size (*F*(1,39) = 117.9, *p* < 0.001, η^2^ = 0.752) and distractor presence (*F*(1,39) = 10.96, *p* = 0.002, η^2^ = 0.219) but no reliable interaction between the two (*F*(1,39) = 3.146, *p* = 0.084; Fig. 2A). Participants were reliably faster when a distractor was present versus when it was absent, a pattern characteristic of feature search (Gaspelin & Luck, 2018a).

A within-subject *t*-test comparing single versus dual target conditions found 100% of participants were faster in two-target trials than in one-target trials (*W* = 0.000, *p* < 0.001, r_rb = 1.000; Fig. 2B), mirroring a pattern associated with serial search (de Waard & Theeuwes, 2026). Next, focusing exclusively on two-target trials, a 2 x 2 x 2 rmANOVA taking target-target compatibility (compatible/incompatible), distractor presence (present/absent), and display size (six- vs. ten-item displays) replicated the de Waard and Theeuwes’ (2026) effects of distractor and display conditions (both *F*s > 6), but also a main effect of target-target compatibility (*F*(1,39) = 10.92, *p* = 0.002, η^2^ = 0.219). Furthermore, an interaction between compatibility and display size was found (*F*(1,39) = 4.36, *p* = 0.043, η^2^ = 0.101; Fig. 2B) with further pairwise testing revealing that target compatibility was only reliable in the six-item search condition (*t*(39) = 2.628, *p* = 0.012, *d* = 0.416; *t*(39) = 0.414, *p* = 0.681, for six- and ten-item conditions, respectively). No further interactions were found (all *F*s < 1).

Lastly, we compared how target-target distance (the distance between the two targets on two-target displays) affected the compatibility effect in line with what was reported in de Waard and Theeuwes (2026). Because six and ten items had different numbers of target-target distance values (up to five in a ten-item search, but only three in a six-item search), we ran separate rmANOVAs with compatibility (compatible, incompatible) distractor presence (present/absent), and target-target distance as factors (Fig. 2C). For both six- and ten-item searches, main effects for target-target distance were found (*F*(2,78) = 9.751, *p* < 0.001, η^2^ = 0.907; *F*(3,117) = 11.05, *p* < 0.001, η^2^ = 0.917, for six- and ten-item search conditions, respectively). No interactions were noted between the main effect of target-target distance and the other factors in either search condition (all *F*s < 1.2 except for compatibility: distance in six-item search [(2,78) = 2.513, *p* = 0.087] and compatibility: distance: distractor interaction in six-item search [*F*(2,78) = 1.707, *p* = 0.188]).

### Discussion

The current experiment used displays identical to those in Stilwell and Gaspelin (2021) and required participants to adopt what is known as the “feature-search mode” to locate a pre-specified target among heterogeneous non-targets. Replicating prior work (Bacon & Egeth, 1994; Leber & Egeth, 2006; Gaspelin et al., 2015, 2017; Stilwell & Gaspelin, 2021), instead of capturing attention, the presence of the salient color distractor resulted in a distractor benefit effect as participants were faster when the distractor was present than when it was absent.

At the same time, the results show a robust display-size effect of roughly 25 ms per item, consistent with serial (inefficient) search (Wolfe, 1994). Search was also faster when two targets were present rather than one, which further supports a serial account, because responses can be made as soon as either target is selected. At the same time, we observed an overall compatibility effect, which is inconsistent with a serial search account. Following the logic of de Waard and Theeuwes (2026; see also Jeong et al., 2025; Lee et al., 2021), a strictly serial search should yield no compatibility effect. Closer inspection of the data suggests, however, that the compatibility effect was driven primarily by the display-size-6 condition; at display size 10, compatibility was absent, consistent with de Waard and Theeuwes (2026). They proposed a “clump-wise” serial search in which attention samples small subsets of items, estimating clump size at display size 10 to be about three to four items. The present finding of target-compatibility effects at display size 6 suggests that, in smaller displays, participants may have been able to use larger clumps and thus process part of the display more in parallel, producing compatibility effects. Importantly, this implies that participants likely switched strategies across trials, adopting a predominantly serial (clump-wise) search for larger displays (display size 10) and a more parallel (or larger-clump) search for smaller displays (display size 6). This trial-to-trial shift was likely driven primarily by display characteristics.

## Experiment 2

Experiment 1 showed no target–target compatibility effect at display size 10, replicating de Waard and Theeuwes (2026). However, when participants searched among six items, a compatibility effect was observed. Experiment 2 tested whether the compatibility effect at small display sizes is a consequence of sparse displays. Specifically, it examined whether larger display sizes would yield serial search, as indicated by a positive set-size slope, without a target–target compatibility effect. Accordingly, in Experiment 2 we used display sizes of eight and 12 items.

### Methods

#### Participants

The primary effect of interest from Experiment 1 was the compatibility effect between two-target conditions. As such we based our sample size in Experiment 2 on the effect size of this comparison (*d* = 0.548), calculating that 40 participants were needed to detect this effect with 95% confidence. As such we used the same sample size as in Experiment 1.

Forty new participants aged between 19 and 40 years (mean age = 30 years) took part in the study through Prolific. Inclusion criteria were identical to those used in Experiment 1. The experiment again took approximately 45 min and participants were paid £6.75. All participants gave informed consent, and all methods were performed in accordance with the Declaration of Helsinki. The experiment was approved by the Ethics Committee of the Faculty of Behavioral and Movement Sciences of the Vrije Universiteit Amsterdam.

#### Procedure

Experiment 2 was nearly identical to Experiment 1 with several notable differences. Most importantly, participants now encountered either 12- or eight-item search arrays instead of ten or six as was the case in Experiment 1(Fig 1). To accommodate the larger 12-item arrays, the radius of the imaginary circle was increased to 230 px. Additionally, due to using 12 items now, the maximum number of each of the five non-target shapes was allowed to be present on each array up to three times, randomly selected. Targets and distractors were prevented from ever appearing directly adjacent to one another, similar to Experiment 1, ten-item conditions.

### Results

**Fig. 3:**
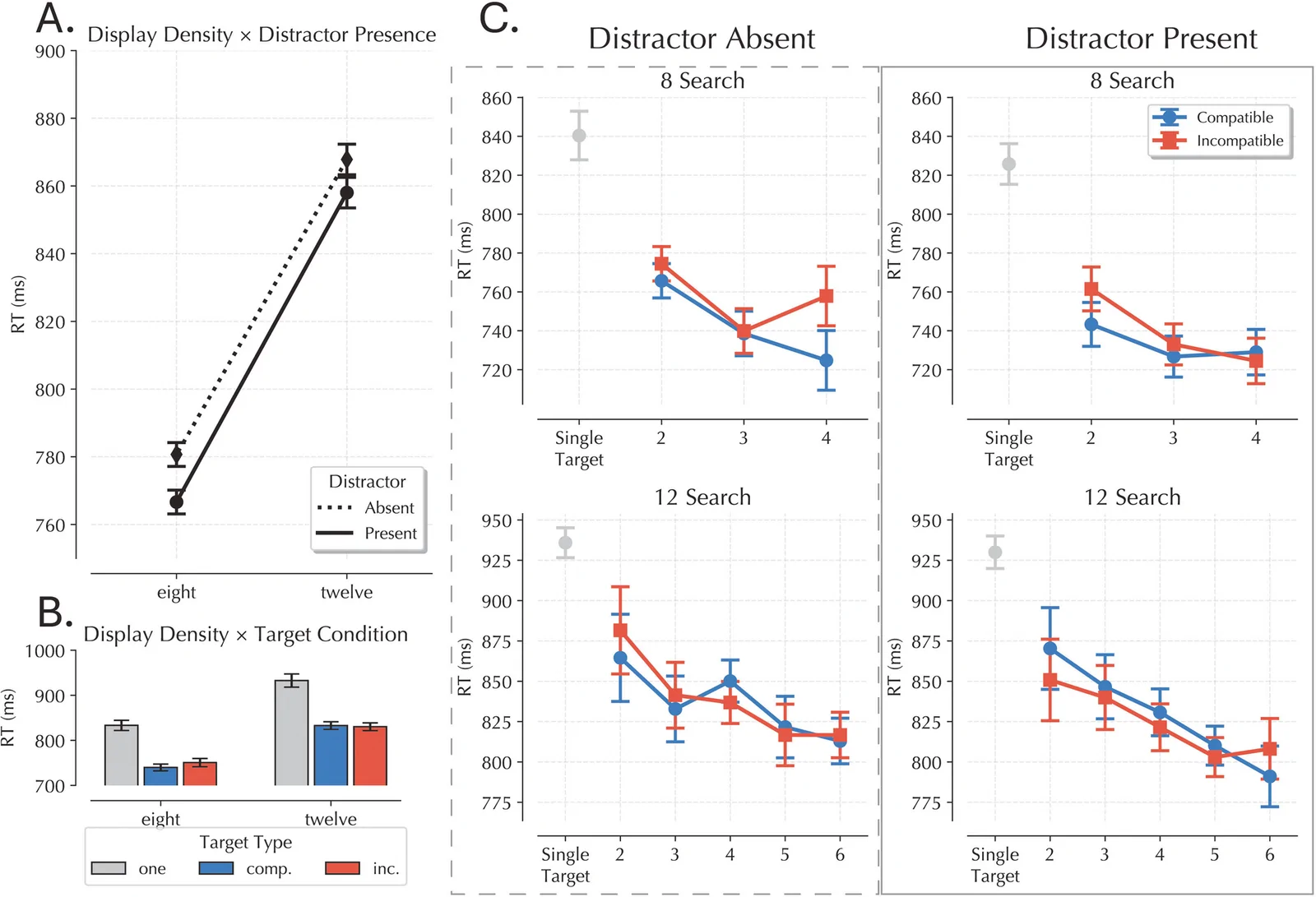
Results from Experiment 2 where search arrays were randomly swapped between eight- and 12-item displays. (**A**) Interaction between number of search items (eight or 12) and distractor condition (present or absent). Targets were detected reliably faster in eight-item arrays than in 12-item displays (~90 ms), but no interaction with distractor presence was found. (**B**) Comparison of target conditions, including two-target compatibility conditions. Single-target trials were reliably slower than both dual-target trials, but that no difference was found between compatible and incompatible target trials. (**C**) Target-target distance effects separated by display density (eight- and 12-item search displays) and distractor-presence conditions (present/absent). Y-axes matched within rows but not columns. All error bars are within-subject 95% confidence intervals (Cousineau, 2005; Morey, 2008)

Excluding trials 2.5 standard deviations away from participant means resulted in the exclusion of 4.7% of the total data. An rmANOVA taking distractor condition and display size (eight and 12) again found significant main effects of distractor presence (*F*(1,39) = 16.61, *p* < 0.001, η^2^ = 0.299) and display size (*F*(1,39) = 173.3, *p* < 0.001, η^2^ = 0.816), but again found no interaction between the two (*F*(1,39) = 0.642, *p* = 0.428; Fig. 3A). A further within-subject *t*-test again found that single-target trials were reliably slower than dual-target trials (W = 0.000, p < 0.001, r_rb = 1.000). Focusing on the dual-target trails, an rmANOVA taking display size, distractor condition, and target-target compatibility as main factors again replicated the above effects of display density and distractor presence (both *F*s > 17), but this time failed to detect a main effect of target-target compatibility (*F*(1,39) = 1.361, *p* = 0.25; Fig. 3B), suggesting that these larger display sizes no longer led to partially parallel search. No further interactions were found (*F*(1,39) = 3.192, *p* = 0.082 for compatibility:display size interaction, all other *F*s < 1).

Separate rmANOVAs for eight- and 12-item search displays taking target-target distance, distractor presence and target-target compatibility as factors, revealed main effects for target-target distance in both display sizes (*F*(2,78) = 9.868, *p* < 0.001, η^2^ = 0.908; *F*(4,156) = 13.5, *p* < 0.001, η^2^ = 0.931, for eight and 12 display sizes, respectively; Fig. 3C), indicating again that responses were slower when targets were separated by more items. Neither search display size observed any interactions between the main effect of target-target distance and any of the other factors (all *F*s < 1.9).

### Discussion

Experiment 2 showed that with larger display sizes there was a clear set-size effect, while no target–target compatibility effect was observed. This pattern provides strong evidence that, in feature-search mode, search is at least partly serial.

## General discussion

The present study set out to link the newly developed target-target compatibility effect to more traditional search slope diagnostics while also providing evidence for (at least partly) serial feature search in commonly used stimulus displays. The results converge on a clear conclusion: First, reliable slopes between display sizes indicate that search in these displays that are assumed to induce feature search is not conducted in parallel. At the same time, the absence of a target–target compatibility effect (at least for the higher set sizes) suggests that the two targets are not processed simultaneously to the level required for response-level interaction. Together, these findings support the idea that when using the feature-search mode, search proceeds at least partly serially, consistent with a clump-wise or item-by-item (serial) selection account. Also, the observation that the two-target search was substantially faster than the single-target search suggests serial search: when two targets are present, the probability of encountering a target earlier in the scan increases, reducing the number of inspected items before a decision can be made.

In the current study, we used the very same displays as those who argue that these displays elicit top-down control, thereby preventing attentional capture by irrelevant distractors (Bacon & Egeth, 1994; Chang & Egeth, 2019, 2021; Gaspelin & Luck, 2018b; Lamy et al., 2004; Leber & Egeth, 2006; Stilwell & Gaspelin, 2021). Consistent with previous findings, we also observed no capture by the salient distractor. Even though some degree of top-down control can never be fully excluded, the more parsimonious explanation is that these displays require at least a partly serial search to locate the target, and that this sequential selection is precisely why capture is reduced or absent. Specifically, the display characteristics necessitate discrimination among individuated items, which promotes (partially) serial search (Liesefeld et al., 2021; Liesefeld & Müller, 2020; Liesefeld & Müller, 2023; Theeuwes, 2004, 2010, 2023a; Wang & Theeuwes, 2020).

As shown previously (Chang & Egeth, 2019, 2021; Gaspelin & Luck, 2018a), instead of observing attentional capture by the salient distractor, we find a distractor-presence benefit: observers are faster when a distractor is present than when it is absent. Many proponents of the idea that feature-search mode reflects top-down control find this result puzzling. For example, Chang and Egeth (2019) argued that “the actual reversal of the capture effect is a striking finding” (p. 1731). However, although many of these authors may consider the effect striking, it is less surprising if one realizes that search is conducted serially (as we propose here), because observers simply have one fewer item to inspect when the distractor is present than when it is absent.

The present findings suggest that when displays are relatively sparse (as in our set-size-six condition), the functional “clumps” in selection become large enough for target–target compatibility effects to emerge. de Waard and Theeuwes (2026) argued that with ten-item displays, selection may proceed in clumps of roughly three to four items. If clump size remains in that range, then reducing set size effectively increases the proportion of the display items processed per selection episode, making it more likely that both targets fall within the same selected subset. This provides a straightforward account of why compatibility effects are more likely to appear in sparser displays. Note, however, that a wide attentional window does not necessarily imply that attention will be captured by the distractor. Sparse displays not only render the target be less salient, the distractor also becomes less salient. Indeed, when only a few elements are equally spaced around fixation, the elements are relatively far apart, thereby reducing its local feature contrast (Nothdurft, 1993). For example, Theeuwes (1992; Experiment 3) showed that even when search proceeds in parallel, as indicated by the absence of a display-size effect, capture was absent when the color singleton distractor was not very salient (see also Wang & Theeuwes, 2020).

While we show here that attentional capture does not occur when search proceeds serially, the study by Leber and Egeth (2006) suggests that under specific conditions, capture can also be prevented when search is presumed to operate in parallel. Their key finding was that, after a training phase with heterogeneous displays designed to induce a feature-search-mode strategy, participants showed no attentional capture during a subsequent test phase using the classic additional-singleton paradigm introduced by Theeuwes (1992). They argued that top-down strategies, such as feature-search mode, can prevent capture even during parallel search. Although these findings are compelling, an alternative possibility is that the training phase encouraged participants to adopt a more serial scanning strategy, which they then continued to use during the test phase, even though the task could in principle have been performed in parallel. However, the data of Leber and Egeth (2006) do not suggest a serial search strategy, as search functions were essentially flat, indicating parallel search. Although this interpretation is post hoc, it is possible that search was in fact serial, but that this was masked by inefficient search at display size 5. Notably, the data reported by Leber and Egeth (2006) show that search in the feature-search-trained group was substantially slower than in the singleton-search-trained group, suggesting that participants did not simply switch to an efficient singleton-detection mode, but instead continued to rely on an inefficient serial search strategy. The observed flat search functions may be related to inefficient search at display size 5. At display size 5, when elements are equally spaced around a central fixation point, they are relatively far apart, thereby reducing local feature contrast (Nothdurft, 1993). This may have impaired serial search at display size 5, thereby increasing search times at this display size and making the search function appear flat, effectively masking a display-size effect.

This interpretation is also supported by a recent paper by Duncan and Theeuwes (under review). In that study, feature-search mode was required on most trials because the target appeared among heterogeneous non-targets. On one-eighth of trials, however, the target could in principle be found by parallel search using singleton detection. The results showed strong contextual persistence: when feature-search trials predominated, search was slow, target–target compatibility effects were absent, and salient distractors failed to capture attention, even on trials in which singleton detection was possible. Thus, once participants had adopted feature-search mode, attentional capture by a salient distractor was prevented.

The current findings fit well with the attentional window account proposed by Theeuwes and colleagues (Belopolsky & Theeuwes, 2010; Belopolsky et al., 2007; Theeuwes, 1994, 2004). Rather than assuming distinct search modes, this account proposes that search depends on the size of the attentional window. When the target is salient, as in the classic additional-singleton task, the window is large and encompasses most of the display, enabling efficient parallel search but also allowing other salient items to capture attention. When the target does not stand out, as in displays assumed to induce feature search, the window becomes smaller and search proceeds more serially across one or a few items at a time (Duncan et al., 2025; Lee et al., 2021; Liesefeld & Müller, 2020). Because processing is largely limited to the current attentional window, distractors outside it are effectively ignored, much like the second target in the present study. It should be noted that, for several reasons, the attentional window account proposed by Theeuwes has been subject to criticism (Gaspelin et al., 2012; Kerzel & Huynh Cong, 2024; Leonard et al., 2013; Ma et al., 2026)

In sum, the present results indicate that in displays commonly assumed to induce feature search, the target cannot be located through parallel processing but instead requires at least partly serial, clump-wise selection. The display-size effects and the pattern of compatibility results support this view, suggesting that observers inspect subsets of items sequentially. Under these conditions, attentional capture does not occur, not because of top-down control, but because observers must discriminate among individuated items.

## Data Availability

Anonymized participant data are available at: osf.io/g6avc

## Code availability

All experiment and analysis code are available at: osf.io/g6avc

## References

[CR1] Bacon, W. F., & Egeth, H. E. (1994). Overriding stimulus-driven attentional capture. *Perception & Psychophysics,**55*(5), 485–496. 8008550 10.3758/bf03205306

[CR2] Belopolsky, A. V., & Theeuwes, J. (2010). No capture outside the attentional window. *Vision Research,**50*(23), 2543–2550. 20807547 10.1016/j.visres.2010.08.023

[CR3] Belopolsky, A. V., Zwaan, L., Theeuwes, J., & Kramer, A. F. (2007). The size of an attentional window modulates attentional capture by color singletons. *Psychonomic Bulletin & Review,**14*, 934–938. 18087962 10.3758/bf03194124

[CR4] Chang, S., & Egeth, H. E. (2019). Enhancement and suppression flexibly guide attention. *Psychological Science,**30*(12), 1724–1732. 31693453 10.1177/0956797619878813

[CR5] Chang, S., & Egeth, H. E. (2021). Can salient stimuli really be suppressed? *Attention, Perception, & Psychophysics,**83*(1), 260–269. 10.3758/s13414-020-02207-8 33241528

[CR6] Cousineau, D. (2005). Confidence intervals in within-subject designs: a simpler solution to Loftus and Masson’s method. *Tutorials in quantitative methods for psychology*, 1(1), 42–45.

[CR7] de Waard, J., & Theeuwes, J. (2026). Beyond top-down: Feature search as a serial clump-wise process. *Cognition,**266*, 106334. 41016254 10.1016/j.cognition.2025.106334

[CR8] Duncan D.H. & Theeuwes, J. (under review). Switching between the singleton detection and feature-search mode in visual search.

[CR9] Duncan, D. H., Van Moorselaar, D., & Theeuwes, J. (2025). Visual statistical learning requires attention. *Psychonomic Bulletin & Review,**32*(3), 1240–1253. 39497006 10.3758/s13423-024-02605-1 PMC12092514

[CR10] Duncan, D. H., van Moorselaar, D., & Theeuwes, J. (2026). Learning alters salience and proactive attentional priority. *Communications Psychology,**4*, 57. 41703279 10.1038/s44271-026-00411-0 PMC13021526

[CR11] Egeth, H. E., & Yantis, S. (1997). Visual attention: Control, representation, and time course. *Annual Review of Psychology,**48*(1), 269–297. 9046562 10.1146/annurev.psych.48.1.269

[CR12] Folk, C. L., Remington, R. W., & Johnston, J. C. (1992). Involuntary covert orienting is contingent on attentional control settings. *Journal of Experimental Psychology: Human Perception and Performance,**18*(4), 1030. 1431742

[CR13] Gaspelin, N., Leonard, C. J., & Luck, S. J. (2017). Suppression of overt attentional capture by salient-but-irrelevant color singletons. Attention, perception & psychophysics, 79(1), 45–62. 10.3758/s13414-016-1209-1 10.3758/s13414-016-1209-1 PMC5182083 27804032

[CR14] Gaspelin, N., & Luck, S. J. (2018). Distinguishing among potential mechanisms of singleton suppression. *Journal of Experimental Psychology: Human Perception and Performance,**44*(4), 626. 29035072 10.1037/xhp0000484 PMC5897145

[CR15] Gaspelin, N., & Luck, S. J. (2018). The role of inhibition in avoiding distraction by salient stimuli. *Trends in Cognitive Sciences,**22*(1), 79–92. 29191511 10.1016/j.tics.2017.11.001 PMC5742040

[CR16] Gaspelin, N., Ruthruff, E., Lien, M.-C., & Jung, K. (2012). Breaking through the attentional window: Capture by abrupt onsets versus color singletons. *Attention, Perception, & Psychophysics,**74*(7), 1461–1474. 10.3758/s13414-012-0343-7 22806409

[CR17] Gaspelin, N., Leonard, C. J., & Luck, S. J. (2015). Direct evidence for active suppression of salient-but-irrelevant sensory inputs. *Psychological Science,**26*(11), 1740–1750. 26420441 10.1177/0956797615597913 PMC4922750

[CR18] Gaspelin, N., Ma, X., & Luck, S. J. (2025). Signal suppression 20: An updated account of attentional capture and suppression. *Psychonomic Bulletin & Review,**32*(6), 1–21. 40728695 10.3758/s13423-025-02736-z PMC12313171

[CR19] Jeong, H. C., Jung, K., & Han, S. W. (2025). Color singletons are suppressed under serial search, but not abrupt onsets. *Cognition,**262*, 106173. 40328168 10.1016/j.cognition.2025.106173

[CR20] Kerzel, D., & Huynh Cong, S. (2024). Search mode, not the attentional window, determines the magnitude of attentional capture. *Attention, Perception, & Psychophysics,**86*(2), 457–470. 10.3758/s13414-022-02582-4 PMC10806210 36207666

[CR21] Lamy, D., & Egeth, H. E. (2003). Attentional capture in singleton-detection and feature-search modes. *Journal of Experimental Psychology: Human Perception and Performance,**29*(5), 1003. 14585019 10.1037/0096-1523.29.5.1003

[CR22] Lamy, D., Leber, A., & Egeth, H. E. (2004). Effects of task relevance and stimulus-driven salience in feature-search mode. *Journal of Experimental Psychology: Human Perception and Performance,**30*(6), 1019. 15584812 10.1037/0096-1523.30.6.1019

[CR23] Lange, K., Kühn, S., & Filevich, E. (2015). "Just Another Tool for Online Studies" (JATOS): an Easy Solution for Setup and Management of Web Servers Supporting Online Studies. *PloS one*, 10(6), e0130834. 10.1371/journal.pone.0130834 10.1371/journal.pone.0130834 PMC4482716 26114751

[CR24] Leber, A. B., & Egeth, H. E. (2006). It’s under control: Top-down search strategies can override attentional capture. *Psychonomic Bulletin & Review,**13*(1), 132–138. 16724780 10.3758/bf03193824

[CR25] Lee, J., Jung, K., & Han, S. W. (2021). Serial, self-terminating search can be distinguished from others: Evidence from multi-target search data. *Cognition,**212*, 104736. 33887651 10.1016/j.cognition.2021.104736

[CR26] Leonard, C. J., Lopez-Calderon, J., Kreither, J., & Luck, S. J. (2013). Rapid feature-driven changes in the attentional window. *Journal of Cognitive Neuroscience,**25*(7), 1100–1110. 23448524 10.1162/jocn_a_00376 PMC3859374

[CR27] Liesefeld, H. R., & Müller, H. J. (2020). A theoretical attempt to revive the serial/parallel-search dichotomy. *Attention, Perception, & Psychophysics,**82*, 228–245. 10.3758/s13414-019-01819-z 31321649

[CR28] Liesefeld, H. R., & Müller, H. J. (2023). Target salience and search modes: A commentary on Theeuwes (2023). *Journal of Cognition,**6*(1), 38. 37426059 10.5334/joc.279 PMC10327865

[CR29] Liesefeld, H. R., Liesefeld, A. M., & Müller, H. J. (2021). Attentional capture: An ameliorable side-effect of searching for salient targets. *Visual Cognition,**29*(9), 600–603.

[CR30] Liesefeld, H. R., Lamy, D., Gaspelin, N., Geng, J. J., Kerzel, D., Schall, J. D., Allen, H. A., Anderson, B. A., Boettcher, S., & Busch, N. A. (2024). Terms of debate: Consensus definitions to guide the scientific discourse on visual distraction. *Attention, Perception, & Psychophysics,**86*(5), 1445–1472. 10.3758/s13414-023-02820-3 PMC11552440 38177944

[CR31] Liesefeld, H., Michaelsen, T., & Janczyk, M. (2025). Salient distractors are rapidly rejected during inefficient visual search. *OSF*. 10.31234/osf.io/dmyn7_v1

[CR32] Luck, S. J., Gaspelin, N., Folk, C. L., Remington, R. W., & Theeuwes, J. (2021). Progress toward resolving the attentional capture debate. *Visual Cognition,**29*(1), 1–21. 33574729 10.1080/13506285.2020.1848949 PMC7872136

[CR33] Ma, X., Luck, S. J., & Gaspelin, N. (2026). Ignoring salient distractors inside and outside the attentional window. *Journal of Cognitive Neuroscience,**38*(2), 242–263. 40991724 10.1162/JOCN.a.105

[CR34] Mathôt, S., Schreij, D., & Theeuwes, J. (2012). OpenSesame: an open-source, graphical experiment builder for the social sciences. Behavior research methods, 44(2), 314–324. 10.3758/s13428-011-0168-7 10.3758/s13428-011-0168-7 PMC3356517 22083660

[CR35] Morey, R. D. (2008). Confidence intervals from normalized data: a correction to Cousineau (2005). *Tutorials in quantitative methods for psychology*, 4(2), 61–64.

[CR36] Nothdurft, H.-C. (1993). The role of features in preattentive vision: Comparison of orientation, motion and color cues. *Vision Research,**33*(14), 1937–1958. 8249312 10.1016/0042-6989(93)90020-w

[CR37] Palan, S., & Schitter, C. (2018). Prolific. ac—A subject pool for online experiments. *Journal of Behavioral and Experimental Finance,**17*, 22–27.

[CR38] Palmer, E. M., Horowitz, T. S., Torralba, A., & Wolfe, J. M. (2011). What are the shapes of response time distributions in visual search? *Journal of Experimental Psychology: Human Perception and Performance,**37*(1), 58. 21090905 10.1037/a0020747 PMC3062635

[CR39] Sawaki, R., & Luck, S. J. (2010). Capture versus suppression of attention by salient singletons: Electrophysiological evidence for an automatic attend-to-me signal. *Attention, Perception, & Psychophysics,**72*(6), 1455–1470. 10.3758/APP.72.6.1455 PMC3705921 20675793

[CR40] Stilwell, B. T., & Gaspelin, N. (2021). Attentional suppression of highly salient color singletons. *Journal of Experimental Psychology: Human Perception and Performance,**47*(10), 1313. 34766817 10.1037/xhp0000948

[CR41] Theeuwes, J. (1991). Cross-dimensional perceptual selectivity. *Perception & Psychophysics,**50*(2), 184–193. 1945740 10.3758/bf03212219

[CR42] Theeuwes, J. (1992). Perceptual selectivity for color and form. *Perception & Psychophysics,**51*(6), 599–606. 1620571 10.3758/bf03211656

[CR43] Theeuwes, J. (1994). Endogenous and exogenous control of visual selection. *Perception,**23*(4), 429–440. 7991343 10.1068/p230429

[CR44] Theeuwes, J. (2004). Top-down search strategies cannot override attentional capture. *Psychonomic Bulletin & Review,**11*(1), 65–70. 15116988 10.3758/bf03206462

[CR45] Theeuwes, J. (2010). Top–down and bottom–up control of visual selection. *Acta Psychologica,**135*(2), 77–99. 20507828 10.1016/j.actpsy.2010.02.006

[CR46] Theeuwes, J. (2023). The attentional capture debate: When can we avoid salient distractors and when not? *Journal of Cognition,**6*(1), 35. 37426061 10.5334/joc.251 PMC10327859

[CR47] Theeuwes, J. (2023). The attentional window, search difficulty and search modes: a reply to commentaries on Theeuwes (2023). *Journal of Cognition,**6*(1), 40. 37426060 10.5334/joc.305 PMC10327826

[CR48] Theeuwes, J. (2025). Attentional capture and control. *Annual Review of Psychology,**76*, 251–273. 10.1146/annurev-psych-011624-025340 39401852 10.1146/annurev-psych-011624-025340

[CR49] Theeuwes, J. (2026). 35+ years of the additional singleton task: Design features and guidelines. *Attention, Perception, & Psychophysics,**88*(5), 119. 10.3758/s13414-026-03259-y PMC13124814 42050109

[CR50] Treisman, A. M., & Gelade, G. (1980). A feature-integration theory of attention. Cognitive psychology, 12(1), 97–136. 10.1016/0010-0285(80)90005-5 10.1016/0010-0285(80)90005-5 7351125

[CR51] Wang, B., & Theeuwes, J. (2020). Salience determines attentional orienting in visual selection. *Journal of Experimental Psychology: Human Perception and Performance,**46*(10), 1051. 32757594 10.1037/xhp0000796

[CR52] Wolfe, J. M. (1994). Guided search 2.0 a revised model of visual search. *Psychonomic Bulletin & Review,**1*(2), 202–238. 24203471 10.3758/BF03200774

[CR53] Wolfe, J. M. (2021). Guided search 6.0: An updated model of visual search. *Psychonomic Bulletin & Review,**28*(4), 1060–1092. 33547630 10.3758/s13423-020-01859-9 PMC8965574

